# Potential explanations for the observed cardiovascular risk profile in military personnel: a narrative review

**DOI:** 10.13075/ijomeh.1896.02886

**Published:** 2026

**Authors:** Magdalena Zawadzka, Ewelina Ejchman-Pac, Justyna Marszałkowska-Jakubik

**Affiliations:** 1 Military Institute of Hygiene and Epidemiology, Department of Health Education, Prevention, and Promotion, Warsaw, Poland; 2 Medical University of Lodz, Department of Epidemiology and Public Health, Łódź, Poland

**Keywords:** public health, cardiovascular diseases, risk factors, occupational exposure, military personnel, environmental exposure

## Abstract

Cardiovascular diseases (CVD) remain the leading cause of mortality in Poland and constitute a major public health burden. While traditional risk factors are well described in the general population, less is known about their distribution in specific occupational groups, including military personnel, who may be exposed to unique work-related cardiovascular risk factors. The aim of this narrative review was to compare the prevalence and profile of major modifiable cardiovascular risk factors in the general Polish population and military personnel and to discuss potential mechanisms underlying the observed cardiovascular risk profile in military populations. This descriptive-analytical study was based on secondary data analysis of epidemiological studies and national reports. Cardiovascular risk factors were highly prevalent in both populations. In military personnel, despite health selection and physical fitness requirements, a substantial proportion presented with overweight, dyslipidemia, and elevated blood pressure, often underdiagnosed. Smoking prevalence remained high in some military subgroups, while suboptimal diet and irregular routines contributed to risk accumulation. Additionally, military personnel may be exposed to chronic stress and other harmful environmental factors. The coexistence and interaction of multiple risk factors highlight the need for targeted, occupation-specific prevention strategies. Expanding CVD prevention to include interventions tailored to military service conditions, including environmental exposures and psychosocial stressors, may improve early detection and reduce long-term cardiovascular burden. Addressing cardiovascular health in this group is essential for maintaining both individual health and operational readiness.

## INTRODUCTION

Cardiovascular diseases (CVD) are the leading cause of mortality in Poland, accounting for approx. 43% of all deaths [1,2]. They are also among the main causes of disability and represent a significant burden on the healthcare system and the economy. Particular importance is attributed to conditions leading to heart failure and other cardiovascular complications, which are characterized by high morbidity, a substantial number of hospitalizations, and high healthcare costs. Population aging and the persistent prevalence of risk factors are expected to further increase the burden of CVD [3].

Cardiovascular risk factors can be broadly divided into 2 main groups:

– non-modifiable factors, including age (risk increases with age), sex (higher risk in men), and genetic predisposition;
– modifiable factors, such as hypertension, dyslipidemia, diabetes and other disorders of glucose metabolism, overweight and obesity, tobacco smoking, and low physical activity [4].

Epidemiological studies indicate that cardiovascular risk factors are widespread in the Polish population. Lipid disorders may affect approx. 30% of adults, and a significant proportion of the population is characterized by hypertension, excess body weight, or abdominal obesity. Tobacco smoking and disturbances in glucose metabolism also remain important [5–7].

Population-based studies on lifestyle in Poland suggest that, despite gradual improvements in health awareness, modifiable risk factors continue to represent a significant problem. Adherence to recommendations for a healthy lifestyle varies depending on age, sex, and educational level, and many areas still require targeted intervention [8].

In this context, particular attention should be paid to occupational groups with potentially different or elevated risk profiles. One such group is military personnel, for whom available data remain limited and do not allow for clear comparisons with the general population. The etiology of health problems in this population, often referred to as “diseases in uniform,” is largely systemic. The nature of military service promotes irregular meals and suboptimal dietary patterns, while exposure to stress and psychosocial factors remains high and persistent [9].

Despite the high level of physical fitness required in service, uniformed personnel may be exposed to additional risk factors related to the specific nature of their work environment and duties. These include occupational stress and irregular lifestyles, which may contribute to tobacco use and unhealthy dietary behaviors, as well as exposure to environmental factors such as extreme temperatures, heavy metals, noise exposure, electromagnetic field (EMF) radiation, and vectors of endemic infectious diseases [10].

The health status of military personnel is crucial for maintaining operational readiness, and CVD and their risk factors may impair the ability to perform military duties. Therefore, early identification and targeted interventions are essential both for health prevention and national security. Comparing the profile of cardiovascular risk factors in the general population and among military personnel may provide valuable insights for the development of preventive strategies and risk assessment in this occupational group. Despite increasing interest in military health, the number of comparative studies remains limited. The aim of this study was to compare the prevalence of selected cardiovascular risk factors in the general adult population in Poland and among military personnel.

## METHODS

This study has a descriptive-analytical design and is based on a secondary data analysis of the prevalence of cardiovascular risk factors in the general adult population in Poland and among military personnel of the Polish Armed Forces.

The analysis included epidemiological data derived from scientific publications and reports from public institutions, including data from Statistics Poland (Główny Urząd Statystyczny – GUS) and the report *Health status of the population in Poland and its determinants – 2025* prepared by the National Institute of Public Health – National Institute of Hygiene (NIPH NIH – NRI). Results from studies available in the scientific literature concerning both military and general populations were also taken into account.

The analysis focused exclusively on the most commonly reported modifiable cardiovascular risk factors described in the literature, including hypertension, dyslipidemia, type 2 diabetes, overweight and obesity, tobacco smoking, unhealthy dietary patterns, and low physical activity.

The comparison was based on a descriptive analysis of the prevalence of individual risk factors and a synthesis of available findings for both populations.

## RESULTS

The results were structured according to individual modifiable cardiovascular risk factors, including excess body weight, hypertension, diabetes, abnormal lipid profiles, and tobacco smoking.

The approach to risk factors for coronary heart disease proposed by WHO experts in 1998 distinguished between major factors (including hyperlipidemia, disorders of glucose metabolism, hypertension, and tobacco smoking) and those considered less significant, such as obesity and low physical activity. However, contemporary evidence indicates that all of these factors are closely interrelated and should be considered of major importance. In particular, obesity – especially central (abdominal) obesity – plays a key role, as it promotes the development of atherosclerosis and cardiovascular complications through complex metabolic, inflammatory, and hemodynamic mechanisms [11,12].

### Overweight and obesity

Obesity is characterized by excessive accumulation of adipose tissue not only in subcutaneous depots but also in internal organs, including fat infiltration of the heart [13]. Therefore, obesity is not only a disease in itself but also a major risk factor for CVD, primarily through its contribution to the development of hypertension. An increase in BMI of 5 kg/m^2^ is associated with up to a 49% higher risk of developing hypertension [12,14,15]. Another study demonstrated that individuals with obesity have a 3.5-fold higher likelihood of developing hypertension and that approx. 60% of hypertension cases are attributable to increased adipose tissue mass [16].

Excess adiposity leads to activation of the sympathetic nervous system, metabolic disturbances, and chronic low-grade inflammation, all of which contribute to vascular and cardiac damage. Consequently, obesity also increases the risk of heart failure and coronary heart disease, which constitute a significant health and economic burden [12,14,15].

In the general population, excess body weight is highly prevalent, with both overweight and obesity occurring frequently, particularly among men [6]. Analyses accounting for sex and temporal trends indicate notable differences between men and women. In 2018, overweight was observed in 48.4% of men and 30.8% of women, while obesity affected 16.6% and 14.0%, respectively. By 2022, the prevalence of overweight among men slightly decreased (45.2%), with obesity remaining relatively stable (16.4%). In contrast, among women, an upward trend was observed, with overweight increasing to 36.8% and obesity to 15.8%. These findings suggest that overweight remains more common among men; however, an unfavorable increasing trend is evident among women in recent years for both overweight and obesity [1].

In the WOBASZ II (Wieloośrodkowe Ogólnopolskie Badanie Stanu Zdrowia Ludności, second edition) study, which included a representative sample of adult Poles, the prevalence of obesity was 26.0% [17]. According to Eurostat data for 2024, the prevalence of obesity in Poland is 18.6% [18], while WHO estimates for 2022 indicate that 27.5% of the population is affected [19].

In the military population, the prevalence of overweight is comparable or even higher. In the cardiovascular risk assessment program in military personnel (MIL-SCORE study), overweight was reported in 54.6% of participants and obesity in 14.1%. Among military pilots, the values were slightly lower (overweight 46.0% based on BMI; obesity 17.5%) [20].

A study conducted by Gaździńska et al. [21] at the Military Institute of Aviation Medicine included 1229 Polish Armed Forces personnel from different branches, participating in the National Health Program (Narodowy Program Zdrowia) 2016–2020. The mean (M) BMI in this group was 26.8 kg/m^2^. According to WHO criteria, normal body weight was observed in only 33% of participants, while 49.7% were overweight and 17.3% obese. This indicates that nearly 67% of the studied population had excess body weight, confirming the high prevalence of this issue in the military population.

Similar results were obtained in a subsequent study by Gaździńska et al. [22], of 760 Air Force personnel (including 60 women) participating in the National Health Program 2021–2025. The mean BMI was 26.6 kg/m^2^, with normal body weight observed in 36.6% of participants, overweight in 48.4%, and obesity in 15.0%. These findings indicate that >60% of this highly selected and physically fit occupational group is affected by excess body weight, which may constitute a significant cardiovascular risk factor.

In a 2024 study by Zawadzka et al. [23], 33.8% of military personnel reported overweight, while nearly 15% reported obesity. Moreover, BMI values increased with higher cardiovascular risk categories, confirming the important role of excess body weight as a determinant of overall CVD risk.

These findings suggest that, despite higher physical fitness requirements and periodic medical screening, overweight and obesity remain a significant public health challenge in both civilian and military populations. This may contribute to increased cardiovascular risk and, in the case of military personnel, may also affect operational performance.

### Unhealthy diet

An unhealthy diet is one of the key modifiable risk factors for CVD. Dietary patterns influence the development of CVD both directly and indirectly, through their impact on major risk factors such as hypertension, dyslipidemia, obesity, and disorders of glucose metabolism. Diets high in ultra-processed foods, saturated fatty acids, salt, and simple sugars contribute to the development of these conditions, whereas dietary patterns based on vegetables, fruits, whole grains, and unsaturated fatty acids have cardioprotective effects and are associated with a lower risk of cardiovascular events [24].

In the general Polish population, persistent irregularities in dietary habits are observed, including insufficient consumption of vegetables and fruits (daily intake reported by 37% for fruits and 43% for vegetables) and a high intake of processed foods. Despite gradual improvements in recent years, reported dietary behaviors still deviate from recommended guidelines. Alcohol consumption also remains an important concern – daily alcohol intake is reported by 11% of men and 3% of women. A positive trend, however, is the observed reduction in alcohol consumption, with 27% of Poles declaring complete abstinence, representing an increase of 3 percentage points compared to the previous year and 8 percentage points compared to 2021 [8].

The association between unhealthy dietary habits, including alcohol consumption, and higher cardiovascular risk categories (Systematic Coronary Risk Evaluation 2 – SCORE2 and Polish version of SCORE – Pol-SCORE) indicates that lifestyle factors play a key role in shaping CVD risk, also in the military population. Approximately 43% of military personnel report unhealthy dietary habits. Nearly 20% of respondents reported regular alcohol consumption, occurring twice as frequently in men compared to women (22.06% vs. 12.00%) and more often among civilian military employees than among active soldiers (31.82% vs. 15.49%) [23].

### Low physical activity

Low physical activity is an important risk factor for CVD, affecting the cardiovascular system both directly and indirectly through the exacerbation of other risk factors. Lack of regular physical activity contributes to the development of obesity and metabolic disturbances, such as insulin resistance, elevated blood glucose levels, and dyslipidemia, which further increase the risk of CVD. In contrast, regular physical activity supports the maintenance of a healthy body weight, improves glycemic control and lipid parameters, and reduces the risk of type 2 diabetes and CVD [14,24].

Data from the nationwide WOBASZ II study indicate that regular physical activity (on most days of the week) is reported by only 27.3% of men and 28.3% of women, while lack of or only occasional activity is observed in 56.8% and 55.2% of participants, respectively. Low physical activity is more common among individuals with lower educational levels, smokers, and those living in large urban areas [17]. Results from the National Health Test indicate a gradual improvement in physical activity levels among Poles. Nevertheless, as many as 46% of adults report no engagement in any sport, while the proportion of individuals undertaking regular physical activity is increasing – 29% report ≥2 h of activity per week. Walking is the most common form of physical activity, with 33% of respondents spending ≥30 min/day and 32% between 30 min and 60 min [8].

In the military population, the proportion of individuals reporting low physical activity was 23.12%, with significantly higher prevalence among women than men (42.00% vs. 16.18%) [23]. The MIL-SCORE study showed that the level of physical activity among soldiers decreases with age. Approximately 30% of soldiers >40 years lead a sedentary lifestyle. Occasional physical activity was reported by approx. 12% of soldiers aged ≤40 years and 30% of those aged >40 years. Although regular physical activity (moderate or high) is reported by the majority, its decline in older age groups coexists with an increase in other cardiovascular risk factors, highlighting the importance of maintaining healthy behaviors throughout life in the prevention of CVD [25].

### Hypertension

Hypertension is the most important modifiable risk factor for CVD. It can directly lead to the development of heart failure through chronic cardiac overload, structural remodeling and fibrosis, and progressive impairment of left ventricular function. At the same time, it is a major contributor to the development of atherosclerotic changes [26].

According to Eurostat data for 2021, the prevalence of hypertension in the general population is 26.5% [18]. Data from the Cardiovascular Disease Prevention Program (Profilaktyka Chorób Układu Krążenia – ChUK), implemented in Poland within primary healthcare, indicate a prevalence of 29.4%, corresponding to approx. 11 million individuals, with clear sex differences (approx. 17–20% in men vs. 7–9% in women) [6]. In the WOBASZ II study, the prevalence of hypertension in the general population was estimated at 42.8% [17].

In the military population, the prevalence of hypertension varies but suggests a substantial underestimation of the problem. In the MIL-SCORE study, hypertension was diagnosed in 13.7% of participants; however, blood pressure values consistent with hypertension were observed in as many as 51.8% of individuals, while only 32.2% had normal (non-elevated) blood pressure. The blood pressure values were M = 134.7/83.1 mm Hg. This indicates that, despite a relatively young population (M = 37.8 years), the blood pressure profile in military personnel is unfavorable, with more than half of participants exhibiting undetected and untreated cardiovascular risk [25].

These findings are consistent with the results reported by Zawadzka et al. [27]. In a 2024 study, mean systolic blood pressure values increased with higher cardiovascular risk categories, ranging from approx. 132 mm Hg in the low-risk group to about 157 mm Hg in the very high-risk group. This demonstrates a clear association between the severity of hypertension and CVD risk classification in the military population [27].

### Diabetes and elevated blood glucose

Diabetes is associated with a twofold increase in cardiovascular risk, including coronary heart disease, ischemic stroke, and vascular mortality [28]. Cardiovascular diseases are also the leading cause of death among individuals with type 2 diabetes [29]. The risk of vascular events in diabetes is higher in women and is present in both type 1 and type 2 diabetes [30]. A reduction in glycated hemoglobin (HbA_1c_) by 1% is associated with a 15% relative reduction in the risk of non-fatal myocardial infarction [31].

In the general population, type 2 diabetes represents a growing epidemiological problem, affecting over 3 million individuals. Data from prevention programs indicate an unfavorable upward trend in blood glucose levels over time, suggesting a worsening burden of disorders of glucose metabolism. Among women, the prevalence increased from 11.0% in 2012 to 13.6% in 2021, with a similar upward trend observed in men (from 19% to 22.8%) [6]. The WOBASZ study also indicates a growing problem of glucose metabolism disorders in the adult population: in WOBASZ I (2003–2005), the proportion of individuals with diagnosed diabetes or fasting glucose levels >7 mmol/l was 6.8%, increasing to 9.8% in WOBASZ II (2013–2014) [17].

According to WHO data, presented via the Organisation for Economic Co-operation and Development (OECD) platform, the prevalence of diabetes in Poland is 10.8% [18] for 2024. Among military personnel, the prevalence of diagnosed diabetes is lower; in the MIL-SCORE study, it was reported at 1.0% [20]. This likely reflects selection into military service and the younger age of the population; however, it does not exclude the presence of early-stage glucose metabolism disorders or undiagnosed disease. It should also be noted that type 2 diabetes often remains asymptomatic for many years prior to diagnosis, making it an important public health challenge in both civilian and military populations.

### Dyslipidemia

Dyslipidemia, defined as abnormal levels of lipids in the blood, is one of the key risk factors for CVD. In most cases, it remains asymptomatic until the occurrence of the first cardiovascular event, such as myocardial infarction, stroke, or peripheral arterial disease. Scientific evidence indicates that only approx. 40% of individuals are aware of their condition [32].

In the general population, elevated cholesterol levels are common and increase with age. In the WOBASZ II study, hypercholesterolemia was observed in 61.1% of the Polish population [17]. In the military population, Polish studies indicate a high prevalence of dyslipidemia; for example, up to 59% of soldiers had elevated low-density lipoprotein (LDL) levels, indirectly reflecting high total cholesterol (TC). Among military pilots, abnormal TC values were observed in more than half of individuals with obesity [20]. In the general population, elevated LDL levels and frequently suboptimal high-density lipoprotein (HDL) levels are predominant [6].

In the MIL-SCORE study, the mean concentration of LDL was 128.3 mg/dl and HDL 52.7 mg/dl, while among pilots the values were 109.1 mg/dl and 55.3 mg/dl, respectively. These findings indicate a high prevalence of an atherogenic lipid profile despite health selection [20]. In another Polish study, increasing SCORE risk categories were associated with higher levels of TC (212.6–258.6 mg/dl), LDL (127.8–158.3 mg/dl), and systolic blood pressure (131.7–157.0 mm Hg), while HDL levels decreased (67.1–57.6 mg/dl). These findings further highlight the protective role of HDL in the context of CVD [27].

### Tobacco smoking

Tobacco smoking is another major risk factor for CVD. Alongside unhealthy diet and excessive alcohol consumption, smoking is a key modifiable determinant of cardiovascular risk. These factors often co-occur with alterations in lipid profiles and metabolic disturbances, and share common underlying mechanisms, including the development of insulin resistance, chronic inflammation, and endothelial dysfunction, which promote the initiation and progression of atherosclerosis [16].

Smoking cessation leads to rapid improvements in cardiovascular function and reduces the risk of recurrent cardiovascular events. However, both current and former smokers have a higher risk of cardiovascular mortality, and even 20 years after cessation, the risk remains elevated compared to individuals who have never smoked [33]. Therefore, preventive strategies aimed at completely avoiding smoking initiation – particularly among young adults and recruits – are crucial for long-term cardiovascular risk reduction.

In the general population, tobacco smoking remains a significant cardiovascular risk factor; however, its prevalence may be higher among military personnel. In the MIL-SCORE study, as many as 46.2% of soldiers reported regular smoking, significantly exceeding values observed in the general population [20]. In Poland, the prevalence of smoking among adults is approx. 25%, with a clear downward trend observed in both men (from 36% to 27.3%) and women (from 24% to 18.0%) [6]. Similar figures were reported in the WOBASZ II study, where the proportion of smokers was 25%, compared to 31% in WOBASZ I, confirming a decreasing trend over time [17]. Data from the WHO, presented via the OECD database, indicate an even lower and more optimistic smoking prevalence in Poland of 17.1% [18].

In the military population, results vary depending on the subgroup studied. Among military pilots, the prevalence of smoking was 10.5%, reflecting the impact of health selection and the specific demands of service [20]. In contrast, in the study by Zawadzka [27], smoking was reported by 23.1% of the overall military-related workforce and 19.6% of active soldiers, indicating a lower but still considerable prevalence of this risk factor. Additionally, smoking prevalence increased with higher cardiovascular risk categories, highlighting its significant role in the accumulation of risk factors within the military population [23].

## SCORE

In a study based on the general population participating in a CVD prevention program (2012–2021), the proportion of individuals at high cardiovascular risk (SCORE ≥5%) was significantly higher among men than among women. At the same time, a decreasing trend was observed over time: in men, from 18.7% in 2012 to 13.1% in 2021, and in women, from 0.49% to 0.26%. A parallel decline in mean SCORE values was also reported [6].

In a cross-sectional study of the military population, the proportion of individuals classified as high or very high risk varied substantially depending on the assessment tool used. Using Pol-SCORE, high/very high risk was observed in 33.57% of men and 3.57% of women, whereas with SCORE2 the proportions increased to 66.43% and 17.86%, respectively. These findings clearly indicate that SCORE2 identifies approximately twice as many individuals at high risk, particularly among men [23].

In another study conducted in a military population, the majority of participants were classified as having moderate cardiovascular risk (65%). This study also confirmed a high prevalence of classical risk factors, including hypertension, dyslipidemia, and excess body weight. In the analyzed group of soldiers, the values of parameters associated with cardiovascular risk increased with higher categories on the Pol-SCORE scale [27].

## DISCUSSION

Although military personnel are generally perceived as a physically fit population and undergo regular preventive medical examinations, the findings presented in this review indicate that the prevalence of several classical cardiovascular risk factors remains high. This apparent paradox may be explained, at least in part, by the specific characteristics of military service. Unlike the general population, soldiers are simultaneously exposed to occupational, behavioral, psychosocial, and operational demands that may contribute to the development and accumulation of cardiovascular risk factors.

One of the most important mechanisms is chronic and acute operational stress associated with military duties, combat readiness, training exercises, and deployments. Persistent activation of the sympathetic nervous system and the hypothalamic–pituitary–adrenal axis results in increased blood pressure, elevated heart rate, and prolonged cortisol secretion, all of which promote the development of hypertension, dyslipidemia, insulin resistance, and other metabolic disturbances [34,35]. These mechanisms probably contribute the relatively high prevalence of elevated blood pressure observed even among young and professionally active soldiers.

Another important contributor is sleep deprivation and circadian rhythm disruption resulting from shift work, night duties, operational tasks, and prolonged field exercises. Growing evidence indicates that chronic sleep restriction is associated with an increased risk of hypertension, obesity, type 2 diabetes, and cardiovascular events [36]. Disturbed sleep may also indirectly worsen cardiovascular health through impaired dietary choices, hormonal dysregulation, and reduced recovery capacity.

The relationship between physical activity and cardiovascular health in military populations appears to be complex. Although regular physical activity is one of the strongest protective factors against cardiovascular disease, military personnel are also exposed to periods of extremely intensive physical exertion associated with military training and operational tasks. In susceptible individuals, particularly those with undiagnosed cardiovascular abnormalities, such exertion may transiently increase the risk of acute cardiovascular events [37,38]. This risk may be further amplified when strenuous physical activity is performed under extreme environmental conditions, including high or low ambient temperatures, high humidity, or high-altitude environments. These conditions increase cardiovascular workload through alterations in thermoregulation, vascular resistance, fluid balance, and hemodynamic responses, thereby potentially exacerbating pre-existing cardiovascular risk [39]. At the same time, declining physical activity with increasing age may contribute to the accumulation of traditional cardiovascular risk factors observed in older military personnel.

In addition, soldiers may experience exposure to occupational hazards, including chronic noise, vibration, airborne particulate matter, combustion products, and other pollutants generated during military training and operational activities. Increasing evidence suggests that these exposures may contribute to chronic systemic inflammation, endothelial dysfunction, oxidative stress, and accelerated atherosclerosis [40–42].

Another important aspect of military service concerns nutrition and lifestyle constraints. During field exercises, deployments, and prolonged operational activities, access to regular, balanced meals may be limited. Consequently, military personnel may rely more frequently on highly processed foods, excessive sodium intake, and irregular meal patterns, all of which contribute to obesity, hypertension, dyslipidemia, and other cardiometabolic abnormalities [43–46]. These behaviors may be further reinforced by psychosocial factors such as prolonged separation from family, uncertainty related to operational duties, and high occupational responsibility, which may promote tobacco smoking, unhealthy dietary habits, and excessive alcohol consumption [44].

Taken together, these military-specific characteristics rarely occur in isolation. Rather, they frequently coexist and may act synergistically, accelerating the development of hypertension, dyslipidemia, obesity, metabolic syndrome, and ultimately cardiovascular disease. Therefore, the relatively high prevalence of classical cardiovascular risk factors observed among military personnel should be interpreted not only in the context of individual lifestyle behaviors but also within the broader occupational environment characteristic of military service.

Therefore, cardiovascular risk in military populations should be interpreted as the result of the interaction between traditional modifiable risk factors and the unique occupational demands of military service rather than as the consequence of isolated exposures.

## CONCLUSIONS

Despite health screening and selection procedures, military personnel demonstrate a substantial prevalence of cardiovascular risk factors, often comparable to that observed in the general population. Particularly concerning are the underdiagnoses of hypertension and the high prevalence of overweight and obesity, dyslipidemia, and tobacco smoking, which may interact and contribute to the accumulation of cardiovascular risk. The use of different cardiovascular risk assessment tools (Pol-SCORE vs. SCORE2) revealed significant discrepancies in identifying individuals at high cardiovascular risk, which may affect the accuracy of identifying those requiring preventive interventions.

These findings suggest that, despite being perceived as a physically fit population, military personnel require targeted and timely preventive measures, as well as systematic monitoring of cardiovascular risk factors. Risk assessment in this population should also take into account the specific characteristics of military service, including chronic stress, irregular lifestyles, and exposure to occupational and environmental factors. This indicates that traditional approaches to cardiovascular disease prevention should be expanded to include interventions tailored to military service conditions.

In Poland, professional soldiers are entitled to annual preventive medical examinations in accordance with the provisions of the Act of 27 June 1997 on the Occupational Health Service [47]. These examinations provide a unique opportunity to implement health promotion activities and facilitate the early identification, monitoring, and management of cardiovascular disease risk factors.

Therefore, regular screening for the early detection of hypertension and metabolic disorders should be complemented by comprehensive, multidimensional intervention programs. Such programs should include health education, support for reducing substance use and managing stress, as well as optimization of dietary habits and evidence-based supplementation tailored to physical activity levels and individual health status. Cardiovascular health among military personnel is not solely an individual concern but represents an important public health challenge with direct implications for operational readiness and national security.

### Study limitations

This study has a narrative-analytical character and is based on secondary data analysis, which is associated with important methodological limitations.

The sources included in the analysis differ in terms of data collection methods, definitions of analyzed variables, and characteristics of the studied populations, which limits direct comparisons between the general and military populations. Data on military personnel are derived primarily from cross-sectional studies and often involve selected subgroups (e.g., aviation personnel only), which restricts their representativeness for the entire military population.

An additional limitation is the lack of standardization in the measurement of risk factors such as physical activity, diet, and tobacco smoking, which in many studies rely on self-reported data and may be subject to information bias. For certain clinical parameters (e.g., blood pressure), underestimation or overestimation of their true prevalence is also possible, depending on the measurement methodology applied.

A further important limitation is the difference in demographic structure between the analyzed populations – the military population is considerably younger and predominantly male, which may influence the observed differences in the prevalence of risk factors.

The lack of studies directly comparing civilian and military populations limits the ability to assess changes over time. Therefore, the presented findings should be interpreted with caution as an approximate representation of differences in cardiovascular risk factors, requiring confirmation in prospective studies using standardized methodologies.

## AI USE

AI tools were used only for English translation.

## References

[1] Wojtyniak B, Smaga A, editors. Sytuacja zdrowotna ludności Polski i jej uwarunkowania – 2025. [Internet]. Warszawa: Narodowy Instytut Zdrowia Publicznego PZH – Państwowy Instytut Badawczy; 2025 [cited 2026 Apr 14]. Available from: https://www.pzh.gov.pl/raport-sytuacja-zdrowotna-ludnosci-polski-i-jej-uwarunkowania-2025/.

[2] Mitkowski P, Witkowski A, Stępińska J, Banach M, Jankowski P, Gąsior M, et al. Position of the Polish Cardiac Society on therapeutic targets for LDL cholesterol concentrations in secondary prevention of myocardial infarctions. Kardiol Pol. 2023;81(7-8):818–23. 10.33963/KP.a2023.0162. 37489830

[3] Lelonek M, Pawlak A, Nessler J, Bohdan M, Hryniewiecki T, Władysiuk M. Niewydolność serca w Polsce 2014–2021. Warszawa: Asocjacja Niewydolności Serca PTK; 2023.

[4] Cegłowska U, Burzyńska M, Prejbisz A, Stępińska J, Gellert R, Pinkas J, et al. Incidence and prevalence of registered hypertension in Poland. Pol Arch Intern Med. 2024;134(6):16746. 10.20452/pamw.16746. 38727185

[5] Witkowski A, Legutko J, Mitkowski P. Stanowisko PTK na temat raportu NFZ o chorobie niedokrwiennej serca. [Internet]. Polskie Towarzystwo Kardiologiczne; 2020 [cited 2026 Apr 14]. Available from: https://ptkardio.pl/aktualnosci/521-stanowisko_ptk_na_temat_raportu_nfz_o_chorobie_niedokrwiennej_serca_naklady_na_leczenie_kardiologiczne_spadaja_smiertelnosc_z_powodu_chorob_serca_nadal_jest_najwieksza.

[6] Kubielas G, Lee CS, Wleklik M, Czapla M, Surma S, Jakubiak GK, et al. Trends in cardiovascular risk factors in Poland: results from the comprehensive cardiovascular risk prevention program. BMC Public Health. 2025 Aug 29;25(1):2962. 10.1186/s12889-025-24385-6. PMC12395911 40883723

[7] Sulicka J, Fornal M, Gryglewska B, Wizner B, Grodzicki T. Wybrane czynniki ryzyka chorób sercowo-naczyniowych u pacjentów podstawowej opieki zdrowotnej. Nadciśnienie Tętnicze. 2006;5:370–6.

[8] Zimny-Zając A. Narodowy Test Zdrowia 2025 [Internet]. Warszawa: Ringier Axel Springer; 2025 [cited 2026 Apr 14]. Available from: https://ocdn.eu/medonet/medonet/Narodowy_Test_Zdrowia_Raport_2025.pdf.

[9] Biała Księga. Zdrowie żołnierza – wyzwanie bezpiecznej przyszłości [Internet]. Warszawa: Instytut Rozwoju Spraw Społecznych; 2026 [cited 2026 Apr 15]. Available from: https://irss.org.pl/wp-content/uploads/biala_ksiega_PL_2026_internet.pdf.

[10] Wójcik M, Siatkowski I. The effect of cranial techniques on the heart rate variability response to psychological stress test in firefighter cadets. Sci Rep. 2023 May 13;13(1):7780. 10.1038/s41598-023-34093-z. PMC10183023 37179419

[11] World Health Organization. Cardiovascular diseases (CVDs) [Internet]. Geneva: The Organization; 2025 [cited 2026 Apr 15]. Available from: https://www.who.int/news-room/fact-sheets/detail/cardiovascular-diseases-(cvds).

[12] Czech A, Bernas M, Tatoń J. Sercowo-naczyniowe objawy otyłości. Endokrynol Otył Zab Przem Mat. 2007;3(4):85–94.

[13] Bogołowska-Stieblich A, Tałałaj M. Otyłość a choroby układu sercowo-naczyniowego. Post Nauk Med. 2013;26(5B):19–25.

[14] Stożek-Tutro A, Monica M, Ryś P, Wieczysta A, Janiszewska K. Raport “Choroba otyłościowa – wyzwania społeczne, kliniczne i ekonomiczne” [Internet]. Warszawa: Eli Lilly, HTA Consulting; 2024 [cited 2025 Apr 15]. Available from: https://flo.org.pl/index.php/raporty/.

[15] Rosengren A. Obesity and cardiovascular health: the size of the problem. Eur Heart J. 2021 Sep 7;42(34):3404–6. 10.1093/eurheartj/ehab518. PMC8423455 34379742

[16] Seravalle G, Grassi G. Obesity and hypertension. Pharmacol Res. 2017 Aug;122:1–7. 10.1016/j.phrs.2017.05.013. 28532816

[17] Drygas W, Niklas AA, Piwońska A, Piotrowski W, Flotyńska A, Kwaśniewska M, et al. Multi-centre National Population Health Examination Survey (WOBASZ II study): assumptions, methods, and implementation. Kardiol Pol. 2016 Jul 11;74(7):681–90. 10.5603/KP.a2015.0235. 26620680

[18] Dashboard on the state of cardiovascular health in the EU [Internet]. Paris: OECD [cited 2026 Apr 1]. Available from: https://www.oecd.org/en/data/dashboards/state-of-cardiovascular-health-in-the-eu.html.

[19] World Health Organization. Age-standardized prevalence of obesity (defined as BMI = 30 kg/m^2^) in people aged 18 years and over, WHO estimates (%) [Internet]. Geneva: The Organization [cited 2026 Apr 1]. Available from: https://gateway.euro.who.int/en/indicators/hfa_630-3023-age-standardized-prevalence-of-obesity-defined-as-bmi-30-kgm2-in-people-aged-18-years-and-over-who-estimates/#id=27332.

[20] Gawron BM, Gielerak G. Assessment of the prevalence of cardiovascular risk factors and associated health threats among soldiers of NATO member states. Lek Wojsk. 2025;103(4):271–6. 10.53301/lw/203553.

[21] Gaździńska A, Gaździński S, Jagielski P, Kler P. Body composition and cardiovascular risk: a study of Polish military flying personnel. Metabolites. 2023 Oct 21;13(10):1102. 10.3390/metabo13101102. PMC10608823 37887427

[22] Gaździńska A, Jagielski P, Baran P. The risk of orthorexia and the prevalence of emotional eating behaviours among Polish military flying personnel in relation to body mass index (BMI) and sociodemographic factors. Nutrients. 2024 Feb 28;16(5):682. 10.3390/nu16050682. PMC10934470 38474809

[23] Zawadzka M, Ejchman-Pac E, Kowalska A, Szymański P, Marszałkowska-Jakubik J. SCORE2 Outperforms Pol-SCORE in Detecting Increased Cardiovascular Risk. Pathophysiology. 2025;32(3):45. 10.3390/pathophysiology32030045. PMC12452680 40981104

[24] Bodurka A, Michałowska J. Dieta wspomagająca zdrowie sercowo-naczyniowe – zalecenia żywieniowe według American Heart Association 2021. Forum Zaburzeń Metab. 2021;12(4):156–162.

[25] Gielerak G, Krzesiński P, Piotrowicz K, Murawski P, Skrobowski A, Stańczyk A, et al. The prevalence of cardiovascular risk factors among Polish soldiers: The Results from the MIL-SCORE Program. Cardiol Res Pract. 2020 May 18;2020:1–7. 10.1155/2020/3973526. PMC7251455 32509345

[26] Rosei EA, Muiesan ML, Kiowski W. Nadciśnienie tętnicze a niewydolność serca. Chor Serca Naczyń. 2008;5(1):6–10.

[27] Zawadzka M, Marszałkowska-Jakubik J, Ejchman-Pac E, Pająk-Tarnacka B, Szymański P. Assessing cardiovascular risk among Polish soldiers: insights using the POL SCORE tool. J Clin Med. 2025 Mar 20;14(6):2130. 10.3390/jcm14062130. PMC11943057 40142938

[28] The emerging risk factors collaboration. Diabetes mellitus, fasting blood glucose concentration, and risk of vascular disease: a collaborative meta-analysis of 102 prospective studies. Lancet. 2010 Jun 26;375(9733):2215–22. 10.1016/S0140-6736(10)60484-9. PMC2904878 20609967

[29] Strain WD, Paldánius PM. Diabetes, cardiovascular disease and the microcirculation. Cardiovasc Diabetol. 2018;17(1):57. 10.1186/s12933-018-0703-2. PMC5905152 29669543

[30] Rawshani A, Sattar N, Franzén S, Rawshani A, Hattersley AT, Svensson AM, et al. Excess mortality and cardiovascular disease in young adults with type 1 diabetes in relation to age at onset: a nationwide, register-based cohort study. Lancet. 2018;392(10146):477–86. 10.1016/S0140-6736(18)31506-X. PMC6828554 30129464

[31] Ray KK, Seshasai SRK, Wijesuriya S, Sivakumaran R, Nethercott S, Preiss D, et al. Effect of intensive control of glucose on cardiovascular outcomes and death in patients with diabetes mellitus: a meta-analysis of randomised controlled trials. Lancet. 2009;373(9677):1765–72. 10.1016/S0140-6736(09)60697-8. 19465231

[32] Mach F, Baigent C, Catapano AL, Koskinas KC, Casula M, Badimon L, et al. 2019 ESC/EAS Guidelines for the management of dyslipidaemias: lipid modification to reduce cardiovascular risk. Eur Heart J. 2020;41(1):111–88. 10.1093/eurheartj/ehz455. 31504418

[33] Mons U, Muezzinler A, Gellert C, Schottker B, Abnet CC, Bobak M, et al. Impact of smoking and smoking cessation on cardiovascular events and mortality among older adults: meta-analysis of individual participant data from prospective cohort studies of the CHANCES consortium. BMJ. 2015;350:h1551. 10.1136/bmj.h1551. PMC4413837 25896935

[34] Kivimäki M, Steptoe A. Effects of stress on the development and progression of cardiovascular disease. Nat Rev Cardiol. 2018;15(4):215–29. 10.1038/nrcardio.2017.189. 29213140

[35] Edmondson D, von Känel R. Post-traumatic stress disorder and cardiovascular disease. Lancet Psychiatry. 2017;4(4):320–9. 10.1016/S2215-0366(16)30377-7. PMC5499153 28109646

[36] Jaspan VN, Greenberg GS, Parihar S, Park CM, Somers VK, Shapiro MD, et al. The role of sleep in cardiovascular disease. Curr Atheroscler Rep 2024 Jul;26(7):249–62. 10.1007/s11883-024-01207-5. PMC11192677 38795275

[37] Franklin BA, Thompson PD, Al-Zaiti SS, Albert CM, Hivert M-F, Levine BD, et al. Exercise-related acute cardiovascular events. Circulation. 2020 Mar 31;141(13):e705–36. 10.1161/CIR.0000000000000749. 32100573

[38] Lavie CJ, Ozemek C, Carbone S, Katzmarzyk PT, Blair SN. Sedentary behavior, exercise and cardiovascular health. Prog Cardiovasc Dis. 2019;124(5):799–815. 10.1161/CIRCRESAHA.118.312669. 30817262

[39] Lee JKW, Tan B, Kingma BRM, Haman F, Epstein Y. Biomarkers for warfighter safety and performance in hot and cold environments. J Sci Med Sport. 2023;26:71–8. 10.1016/j.jsams.2022.12.006. 36623995

[40] Münzel T, Sørensen M, Daiber A. Transportation noise pollution and cardiovascular disease. Nat Rev Cardiol. 2021;18(9):619–36. 10.1038/s41569-021-00532-5. 33790462

[41] Rajagopalan S, Al-Kindi SG, Brook RD. Air pollution and cardiovascular disease: JACC State-of-the-Art Review. J Am Coll Cardiol. 2018;72(17):2054–70. 10.1016/j.jacc.2018.07.099. 30336830

[42] Ejchman-Pac E, Rzepka M, Gierlach M, Szymański P, Zawadzka M. Evaluation of knowledge and self-awareness of Polish soldiers regarding exposure to chemical and physical factors in their place of service/work. Healthcare 2024;12(4):450. 10.3390/healthcare12040450. PMC10888362 38391825

[43] Mozaffarian D. Dietary and policy priorities for cardiovascular disease, diabetes, and obesity: a comprehensive review. Circulation. 2016;133(2):187–225. 10.1161/CIRCULATIONAHA.115.018585. PMC4814348 26746178

[44] Gaździńska A, Baran P, Turczyńska M, Jagielski P. Evaluation of health behaviours of Polish Army Soldiers in relation to demographic factors, body weight and type of Armed Forces. Int J Occup Med Environ Health. 2023 Nov 13;36(4):437–49. 10.13075/ijomeh.1896.02114. PMC10691415 37184148

[45] Tomczak A, Anyzewska A, Lepionka T, Bolczyk T, Grudniewski T, Gładysz I, et al. Physical activity and eating habits of Polish Navy soldiers and Border Guard officers – a comparative analysis. Nutrients 2024;16(22):3842. 10.3390/nu16223842. PMC11597716 39599628

[46] Li S, Lear SA, Rangarajan S, et al. Association of sitting time with mortality and cardiovascular events in high-income, middle-income, and low-income countries. 2022;7;(8):796–807. 10.1001/jamacardio.2022.1581. PMC9201743 35704349

[47] Ustawa o służbie medycyny pracy. Obwieszczenie Marszałka Sejmu Rzeczypospolitej Polskiej z dnia 13 stycznia 2022 r. w sprawie ogłoszenia jednolitego tekstu ustawy o służbie medycyny pracy. Dz.U. 2022, poz. 437.

